# A Fast Indoor/Outdoor Transition Detection Algorithm Based on Machine Learning

**DOI:** 10.3390/s19040786

**Published:** 2019-02-14

**Authors:** Yida Zhu, Haiyong Luo, Qu Wang, Fang Zhao, Bokun Ning, Qixue Ke, Chen Zhang

**Affiliations:** 1 School of Software Engineering, Beijing University of Posts and Telecommunication, Beijing 100876, China; dozenpiggy@bupt.edu.cn (Y.Z.); bokunning@gmail.com (B.N.); keqixue1997@126.com (Q.K.); ssezc@bupt.edu.cn (C.Z.); 2 Beijing Key Laboratory of Mobile Computing and Pervasive Device, Institute of Computing Technology Chinese Academy of Sciences, Beijing 100190, China; 3 School of Information and Communication Engineering, Beijing University of Posts and Telecommunication, Beijing 100876, China; wangqu@ict.ac.cn

**Keywords:** machine learning, quickly switching, GNSS measurements, indoor/outdoor detection, seamless indoor and outdoor navigation and positioning, smartphone

## Abstract

The widespread popularity of smartphones makes it possible to provide Location-Based Services (LBS) in a variety of complex scenarios. The location and contextual status, especially the Indoor/Outdoor switching, provides a direct indicator for seamless indoor and outdoor positioning and navigation. It is challenging to quickly detect indoor and outdoor transitions with high confidence due to a variety of signal variations in complex scenarios and the similarity of indoor and outdoor signal sources in the IO transition regions. In this paper, we consider the challenge of switching quickly in IO transition regions with high detection accuracy in complex scenarios. Towards this end, we analyze and extract spatial geometry distribution, time sequence and statistical features under different sliding windows from GNSS measurements in Android smartphones and present a novel IO detection method employing an ensemble model based on stacking and filtering the detection result by Hidden Markov Model. We evaluated our algorithm on four datasets. The results showed that our proposed algorithm was capable of identifying IO state with 99.11% accuracy in indoor and outdoor environment where we have collected data and 97.02% accuracy in new indoor and outdoor scenarios. Furthermore, in the scenario of indoor and outdoor transition where we have collected data, the recognition accuracy reaches 94.53% and the probability of switching delay within 3 s exceeds 80%. In the new scenario, the recognition accuracy reaches 92.80% and the probability of switching delay within 4 s exceeds 80%.

## 1. Introduction

Indoor/outdoor (IO) context sensing plays a vital role for numerous applications, for example, human localization and tracking [1,2,3,4,5], activity recognition [6,7] and transportation mode [8,9,10], power management and medical care [11]. For seamless positioning and navigation [12,13], IO detection is a bridge between indoor and outdoor localization. To improve positioning accuracy and reduce power consumption, multi-source fusion positioning system triggers specific positioning and fusion strategies according to the results of IO detection. Smartphones automatically adjust screen brightness according to the IO status and environment condition (e.g., time, weather). The IO status provides personalized service such as adaptively adjusting the device volume. 

According to World Bank statistics, the number of current smartphone users is 4.57 billion, and this is expected to grow to 4.78 billion by 2020 [14]. Recently smartphones are equipped with a variety of sensors as well as powerful processor and storage capabilities. The smartphone-based IO detection mainly benefits from the extensive use of smartphones—consumers always carry smartphones. To provide context-aware information, a lot of previous methods focused on IO detection in a variety of environments have done. The methods are classified into two categories: fixed detection rules or thresholds-based techniques, and machine learning-based techniques.

The first category uses fixed detection rules and thresholds such as a sensor reading above a certain value are considered a state. Zhou et al. [15] and IODetector [16] leveraged smartphone built-in sensors including proximity, light sensor, accelerometer, magnetometer, and cell tower RSS to distinguish between outdoor, semi-outdoor and indoor environments. These were two similar related works, as they utilized hard thresholds in light detector, cellular detector and magnetism detector, and then fused the detection results of each sub-module by Hidden Markov Model (HMM) [17], and achieved a recognition accuracy of above 88% and 92%, respectively, in their campus and city areas. Both mentioned the above algorithm depends on the measurements of a large number of visible neighbor cellular towers. However, most current smartphones do not support recording measurements from all neighboring cellular towers. Zou et al. [18] presented an IO detection technique that leveraged the low power iBeacon technology to discriminate between semi-outdoor and indoor environments. Their test environment is the beacon placement environment on campus and the recognition accuracy reached 96.2%. Li et al. [19] presented a lightweight IO detection based on Wi-Fi RSS signals and a light sensor. The Wi-Fi sub-detector utilized AdaBoost [20] and the light sensor module utilized the threshold to detect the environment separately, and then a semi- conditional random field (CRF) algorithm was used to aggregate the Wi-Fi and light sensor results. The evaluation results showed that the IOS detector can achieve over 96% accuracy in the FIT Building at Tsinghua University, Xidan Street, and CETC office building environments. SatProbe [21] only used the number of GPS visible satellites as a more direct indicator of IO status. They collected 79 segments of raw GPS traces, with 2595 randomly sampled points for detection test and the overall detection accuracy of SatProbe is 85.6%. Gao et al. [22] extracted the number of visible satellites that CNR more than 25 dB-Hz and the sum of all visible satellites CNR that more than 25db-Hz based on the availability and strength of GNSS signals (GPS and GLONASS) as features. Then, a Hidden Markov Model was used to infer the current environment types (indoor, intermediate and outdoor) according to those extracted features and the proposed environmental context detection method is tested in the city of London as a whole, achieving an overall 88.2% accuracy. Meanwhile, GNSS signals received from GNSS receivers had also been used for IO detection [23]. SenseIO [24] designed a ubiquitous multi-model system to fuse cell tower, Wi-Fi-based, activity recognition and light intensity data based on fixed detection rules for IO detection and their experiments for each module and all framework scenarios show that the SenseIO provides promising detection accuracy (above 92%). In [25], utilizing the fixed rules, light sensor, magnetic sensor and satellite signals were integrated to identify the IO status to help achieve seamless indoor and outdoor positioning. However, fixed detection rules or threshold-based methods are difficult to adapt to different environments and devices.

In the second category, features are extracted from smartphone embedded sensors and detected IO status by the machine learning algorithm. SenseMe [26] utilized C4.5 algorithm on data generated from GPS, gyroscope, accelerometer and the Bluetooth module sense environmental context, as well as the context-aware location. They evaluated SenseMe against several metrics with the aid of 2 two-week long live deployments involving 15 participants and the detection accuracy reached 91.23%. Sung et al. [27] proposed a sound-based IO detection method that utilized acoustic features created by different patterns of reverberations according to ambient environments. Then, Sung leveraged a binary classification method to determine the IO environments by using the acoustic feature. Considering the electromagnetic environments are different, the data characteristics of the magnetic sensors under IO situations are different. The experiments were conducted at the KAIST campus located in Daejeon, South Korea and the best accuracy (96.79%) was achieved when the score calculation range was 50, and the threshold value, 2000. In addition, the transition time of their method took only 3.81 s on average. Canovas et al. [28] employed a binary classification technique on the received signal strength indicator (RSSI) from 802.11 access points to identify a pedestrian’s indoor or outdoor status. They conducted experiments on their campus, with a mean error rate around 2.5%. MagIO [29] utilized machine learning algorithms including Support Vector Machines (SVM), Gradient Boosting Machines (GBM), Random Forest (RF), K-Nearest Neighbor (kNN) and Decision Trees (DT) to deal with magnetic signals for IO detection. Experiments showed that Naive Bayes and random forest possess the capability to achieve an accuracy of 80% and higher with magnetic data alone. An ensemble-based stacking approach is presented, as well, which achieves an accuracy of 85.30% for a campus area, shopping mall and subway station using three different smartphones. Wang et al. [30] applied a machine learning algorithm to classify the neighboring GSM station’s signal in different environments and identify the users’ current context by signal recognition. They test the algorithm in four different environments in their campus. The results show that their algorithm is capable of identifying open outdoors, semi-outdoors, light indoors and deep indoors environments with 100% accuracy using four nearby GSM stations’ signal strength. Radu [31] considered employing co-training according to the feature of light, magnetic and cell sensors for detection. It can automatically learn characteristics of new environments and devices and thereby provides a detection accuracy exceeding 90% even in unfamiliar circumstances. Anagnostopoulos [32] leveraged J48 and other machine learning algorithms to detect the IO state. They utilized multiple contextual features such as activity, barometric, ambient light, GSM, magnetometer variance, etc. Using all sensors; they could achieve 99% classification accuracy with a 10-fold cross-validation test. Wi-FiBoost [33] was designed to utilize AdaBoost [20] determines in a fast and accurate way whether a device is inside or outside particular buildings. They conducted all their experiments in two facilities located on their campus and showed that the resulting performance, a mean error rate around 2.5%. Some of the mentioned above algorithm depend on the measurement of signals from a large number of visible neighbor cellular towers. However, most of the current smartphones do not support recording measurements from all neighbor cellular towers. Also, in the mentioned above algorithms, all the algorithms except Sung et al. [27] did not evaluate the indoor and outdoor scene switching delay. 

On the other hand, it is difficult to obtain satisfactory classification results with only two classification labels, since a variety of signal variations in complex scenarios and the similarity of indoor and outdoor signal sources in IO transition regions. Due to the GNSS signal, light intensity, geomagnetism, Wi-Fi and other sensor features are different in the open outdoors and deep indoors. Distinguishing IO state in this two environment is easy. However, the actual indoor and outdoor scenes encountered in the urban area are not all the above two ideal scenes, such as on the overpass, near the tall buildings, inside the glass curtain wall, close to the indoor patio, etc. We define these ideal and non-ideal indoor and outdoor environments as complex scenes. By defining the categories of complex scenes, we present the diversity of scenes for data collection.

**Figure 1:**
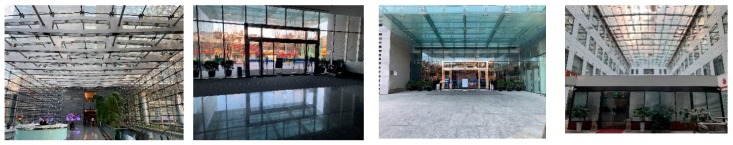
Indoor and outdoor transition areas.

For the mentioned above reasons, a lot of previous studies proposed an ambiguous state like semi-outdoor or shallow indoors to obtain better experiments result. Both fixed detection rules or threshold-based methods achieve satisfactory detection accuracy in ideal open outdoors or closed indoors environments, however, the IO detection accuracy of the abovementioned methods significantly decreases or they are even unable to identify the IO transition areas as shown in Figure 1. However, an uncertain status like semi-outdoor and shallow indoors is difficult for many applications to interpret since the environmental characteristics there are not defined. In this paper, we focus on these complex scenarios, but the final detection status only includes indoors and outdoors. Furthermore, the accuracy and transition delay of IO transition delay in complex scenarios is also a problem we are concerned with.

To accomplish IO detection in complex scenarios, we must address the following two major challenges. We define the scenes without data collection in the training stage and the scenes without specific parameters as new environments. First, the poor performance of IO detection in the new environment is due to a variety of signal variations, since users may be in new scenarios that not match the training phase, such as in a room, near a window, under an overpass, in the open outdoors and so on. Detecting all environments with fixed rules and constraints is impractical. Second, it is difficult to detect the transition between indoor and outdoor environments correctly within a short time due to the similarity of indoor and outdoor signal sources when switching between indoors and outdoors in complex scenarios.

To address both challenges mentioned above, we leverage Global Navigation Satellite System (GNSS) measurements from Android smartphones to detect IO complex environments. Because of the availability and accuracy of satellite signals tend to be less affected by factors other than the environment, we extract spatial geometry distribution, time sequence and statistical features from the GNSS measurements through Android smart mobile devices. Then, we applied supervised machine learning algorithms to predict IO status. Finally, we regard the predicted IO status as the observations of Hidden Markov Model (HMM) [17] to accurately recognize IO status and immediately detect the transition between IO in complex scenarios. To the best of our knowledge, this paper is the first that uses a stacking model with HMM for IO detection.

The main contributions of our work are summarized as follows:

1) We propose a novel IO detection algorithm employing an ensemble model based on stacking. To further filter the occasional detection errors and improve the reliability of IO detection in complex scenarios, we adopt the HMM to the detection results obtained by the ensemble model.
2) We focus on IO switching detection to guarantee the continuity of IO detection. To improve the IO detection accuracy and reduce IO switching delay, we analyze and extract spatial geometry distribution, time sequence and statistical features of GNSS measurements using different sliding windows in Android smartphone rather than other GNSS receivers.
3) Also, to evaluate the proposed algorithm in typical IO scenarios, we compare our proposed algorithm with two state-of-the-art IO detection methods using GNSS information on four different datasets. The experimental results showed that, in the complex IO scenarios, our proposed algorithm achieved higher IO detection accuracy and lower switching delay than other algorithms under the new test environments.

## 2. Materials and Methods

### 2.1. System Overview

**Figure 2:**
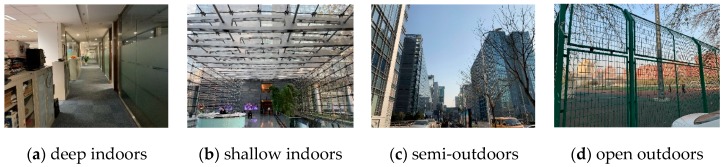
Four types of complex indoor and outdoor scenarios. These four types of scenarios cover campuses, office buildings, shopping malls, overpasses within the city.

We divided complex environments into four types: deep indoors, shallow indoors, semi-outdoors and open outdoors, as shown in Figure 2. Deep indoors is the area far from windows, balcony and glass curtain wall, while shallow indoors is the area opposite of deep indoors. Semi-outdoors is a semi-open area covered by modern buildings, overpasses and patios, while open outdoors is in the non-covered area with better sky exposure. 

**Figure 3:**
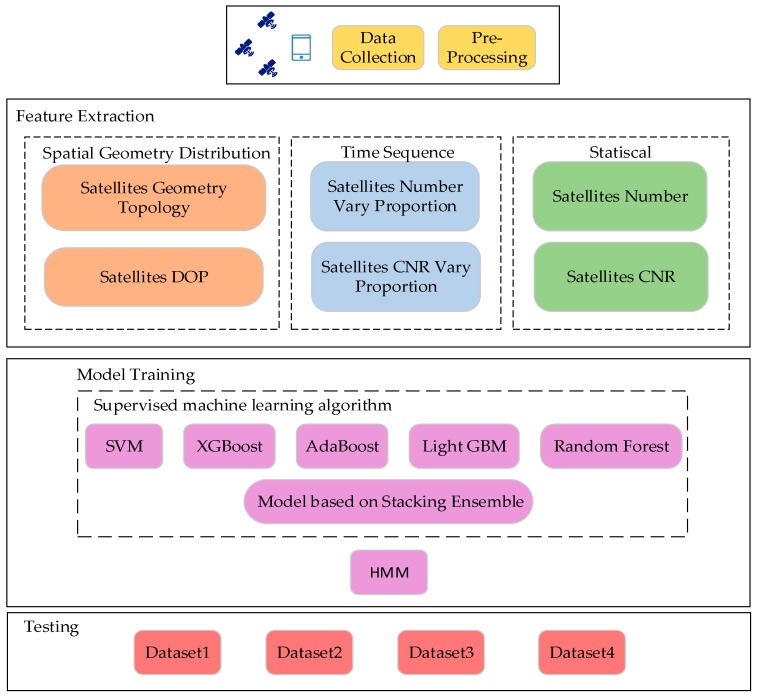
Algorithm framework of our proposed IO detection.

We constructed a dataset by associating the GNSS measurements from Android smartphones and the truth value of the abovementioned scenarios. We extracted 36 features under three types of spatial geometry distribution, time sequence and statistical from GNSS measurements. Depending on the scenarios, the indoors and outdoors scenarios were marked as positive sample and negative sample, respectively. Thus, the IO detection task was transformed into a supervised binary classification problem. Multiple single classification models and a classification model based on stacking were used for training and testing. After the classification model forecast, a hidden Markov model inferred the current environment types from the predictions as final detection results. We evaluated the accuracy and context switch latency of our proposed algorithm under complex scenarios using four different datasets. Figure 3 illustrates the algorithm framework of the proposed IO detection.

### 2.2. Data Collection

GNSS measurements were collocated at 1Hz using different Android smartphones. According to GNSS measurements, we define G as follows:

(1) $$G≜\left\{g_{i}|i=0,1,\cdots ,n\right\},g_{i}≜\left\{ctype_{i},svid_{i},cnr_{i},azi_{i},ele_{i}\right\}$$

where G denotes a set of n visible satellites smartphones recorded in a second. $g_{i}$ denotes visible satellite information, including the constellation type, ID in the constellation, Carrier Noise Ratio(CNR), azimuth and elevation when available.

The datagram information (including $GPGGA, $GPGSA, $GPGSV, and other datagrams) in the NEMA-0183 protocol was obtained through the interface provided by the Android system. We define S as follows:

(2) S≜{pdop,hdop,vdop}

where S contains the value of Position Dilution of Precision (PDoP), Horizontal Dilution of Precision (HDoP), and Vertical Dilution of Precision (VDoP) of the current environment by parsing the $GPGSA datagram from NEMA-0183 protocol.

### 2.3. Data Pre-Processing

**Figure 4:**
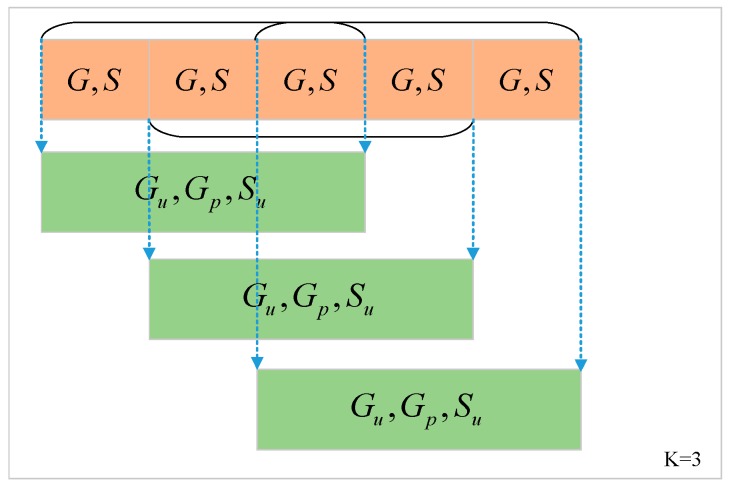
An example of the new GNSS measurements set in the collection sequence when the sliding window length *k* is three.

Environmental change is a continuous process and we need to consider how signal changes within a period for context awareness. Therefore, we introduced a sliding window that contained more descriptive information to preprocess data. Sliding window with different size covers diverse information to detect environment. Figure 4 shows the new GNSS measurements set in the collection sequence when the sliding window length *k* is 3. When the window size *k* is 1, the set of GNSS information G and S are used to extract features of the current environment. When the window length *k* is greater than 1, sliding window obtain time sequence information of GNSS measurements, we define three new sets of GNSS measurements $G_{u}$, $G_{p}$ and $S_{u}$ as follows:

(3) $$G_{u}≜\overset{k}{\underset{i=1}{\cup }}G_{i}=\left\{g_{i}|i=0,1,\cdots ,n\right\},G_{p}≜\overset{k}{\underset{i=1}{\cap }}G_{i}=\left\{g_{i}|i=0,1,\cdots ,n\right\}$$

(4) $$g_{i}≜\left\{ctype_{i}^{},svid_{i}^{},cnr_{i}^{j},azi_{i}^{j},ele_{i}^{j}|j=1,2,\cdots k\right\}$$

(5) $$S_{u}=\left\{pdop_{j},hdop_{j},vdop_{j}|j=1,2,\cdots k\right\}$$

where $G_{u}$ represents the union set and $G_{p}$ represents the intersection set of the G according to the visible satellite constellation and the ID in the constellation as the unique identification when the window length is *k*. $S_{u}$ represents the set of all values of S when the window length is *k*.

The sliding window plays two important roles in the data pre-proposal stage. The first is to reduce the noise fluctuation. Since the GNSS signal received by smartphones is unstable, we calculate the statistical features at the current moment in set $G_{p}$ and $S_{u}$. The other one is to obtain more GNSS information to extract time sequence features. The information of a single moment can hardly reflect the change of signal in time sequence. We utilize different sliding window size (e.g., 2, 3, 5) to smooth the current time GNSS signal and extract time sequence features at different time intervals. In our experiments, we compared different kinds of features in different sliding window size (e.g., 2, 3, 5) in Section 3.2.1.

### 2.4. Feature Extraction

Feature selection affects significantly to the performance of scenario recognition classifier. Table 1 describes the 36 kinds of features extracted by GNSS measurements.

#### 2.4.1. Visible Satellite Geometry Topology

The satellite azimuth is based on the standard of the direct north direction (about the geomagnetic South Pole). The satellite antenna points to the east or west to adjust an angle, which reflects the position of the satellite in space. Its value ranges from 0 to 360°.

**Figure 5:**
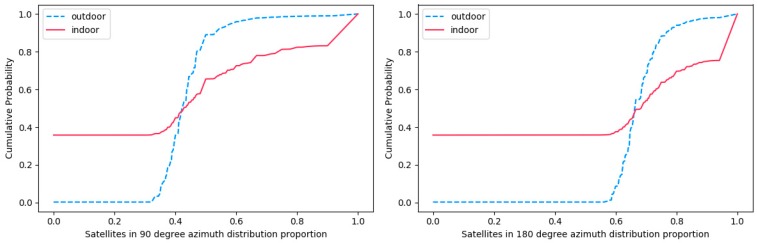
The cumulative probability of the number of satellites in the distribution of satellite azimuth using different smartphones in the range of different angles in a week.

Figure 5 shows the cumulative probability of the number of visible satellites azimuth using different smartphones in the range of 90 and 180° in a week. In outdoor scenarios, within the range of 90°, the ratio of the number of the satellite is mainly concentrated in the range from 0.4 to 0.6. Meanwhile, within the range of 180°, the ratio of the number of the satellite is mainly concentrated in the range from 0.6 to 0.8. However, in indoor scenarios, the ratio of the number of satellite start from 0 due to there is no signal. Furthermore, the ratio of the number of the satellite is mainly concentrated in the range from 0.8 to 1.

We deeply analyzed and compared the distribution of satellite azimuth in complex indoor and outdoor scenarios. In deep indoors, it is difficult to receive the visible satellite signal because the reinforced concrete and the wall structure block the satellite signal. In shallow indoors, through satellite signals may penetrate the glass curtain wall and window, the azimuth range of observable satellites is limited due to limited exposure to the sky. In semi-outdoors, although blocked by tall buildings and overpass, the scope of the exposed sky is larger than that in shallow indoors, the satellite signal can still be detected after reflecting by the multi-path effect. In the open outdoors, the visual satellite is dispersed in the sky with a strong signal. 

**Figure 6:**
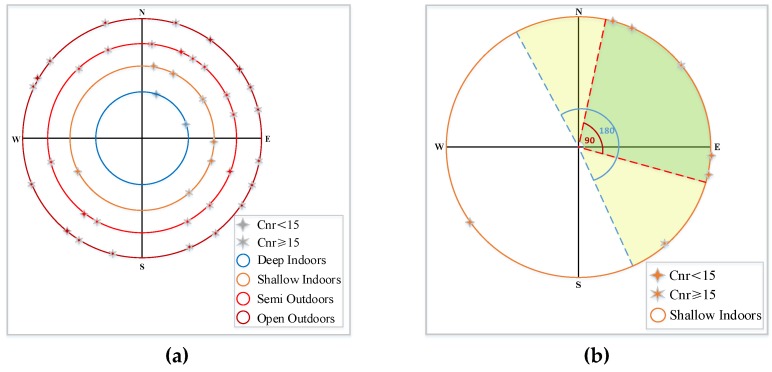
Visible satellite geometry topology (**a**) An example of sky plot and availability of visible satellites under complex scenarios using Huawei Mate 9; (**b**) A sketch demonstrates the distribution of the maximum number of satellites within the range of 90° and 180° in the office window environment.

In the open outdoors, the visual satellite is dispersed in the sky with a strong signal. Figure 6a demonstrates an example of sky plot and availability of visible satellites under complex scenarios using a Huawei Mate 9 smartphone. Figure 6b is a sketch that demonstrates the distribution of the maximum number of satellites within the range of 90° and 180° in the indoor environment with French windows. Based on the above analysis, we extracted the geometric topological features of the visible satellite in set G. We divided the range of azimuth from 0 to 360° into 36 sectors at an interval of 10°. Then, we constructed a 36-dimensional feature vector d to represent the distribution of satellites in these sectors. The vector elements include 0 and 1. 0 indicates no visible satellites in this sector. 1 indicates visible satellites in this sector. The proportion of sector occupied by visible satellites was considered as one feature as follows:

(6) $$A_{z}\_\mathrm{dtb}\_\mathrm{proportion}=\frac{\sum_{i=1}^{36}d_{i}}{36}$$

To further explore the topological relationship of satellites in spatial, the maximum proportion of the number of satellites within the range of 90° and 180° of the azimuth variation of visible satellites in G is extracted as features. We defined a function $f(i,az_{j})$ that calculated whether a satellite is within the range of satellite azimuth. The formula for calculating the GS_num_proportion_range is as follows:

(7) $$f(i,az_{j})=\left\{ \begin{array}{l} 1,\;\mathrm{if}\;i\leq az_{j}\leq \min(i+range,360)\;\mathrm{or}\;az_{j}\leq i+range-360;\\ 0,\;\mathrm{otherwise}. \end{array}\right.$$

where range represents the range of satellite azimuth.

(8) $$\mathrm{GS}\_\mathrm{num}\_\mathrm{proportion}\_\mathrm{range}=\underset{i}{\max}\frac{\sum_{j=1}^{n}f(i,az_{j})}{n},\;0\leq i\leq 360$$

#### 2.4.2. Dilution of Precision for Positioning Satellites

**Figure 7:**
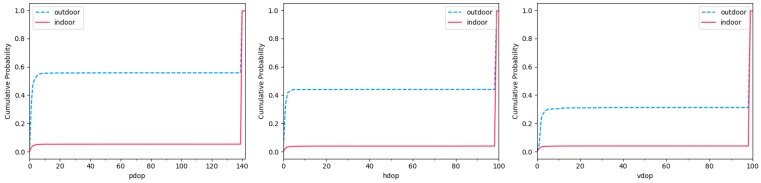
The cumulative distribution probability of the number of satellites in the distribution of satellite azimuth using different smartphones in the range of different angles in a week.

In GPS navigation and positioning, Dilution of Precision (DoP) is used to evaluate the influence of the spatial geometric distribution of visible satellites on positioning accuracy. DoP is an indicator of position quality. We predict the result of position accuracy according to the position relationship of each satellite and other satellites in the constellation. A small DoP value indicates a high probability of strong satellite geometric position and accuracy. We calculated the cumulative distribution probability of DoP values in the data collected in a week under complex indoor and outdoor scenarios, as shown in Figure 7.

In rare cases, there are lower DoP values in an indoor environment. Although there is a certain amount of error in relying on GPS positioning in outdoor scenarios, the value of DoP is generally low. Since it takes a short time for us to collect each piece of data, and smartphones obtain the DoP value through a calculation process, there is a high probability that the DoP value in outdoor scenarios exceeds 10 in our statistical results. We extracted PDoP, HDoP andVDoP as features from S.

#### 2.4.3. The Number of Visible Satellites Vary in Weight

**Figure 8:**
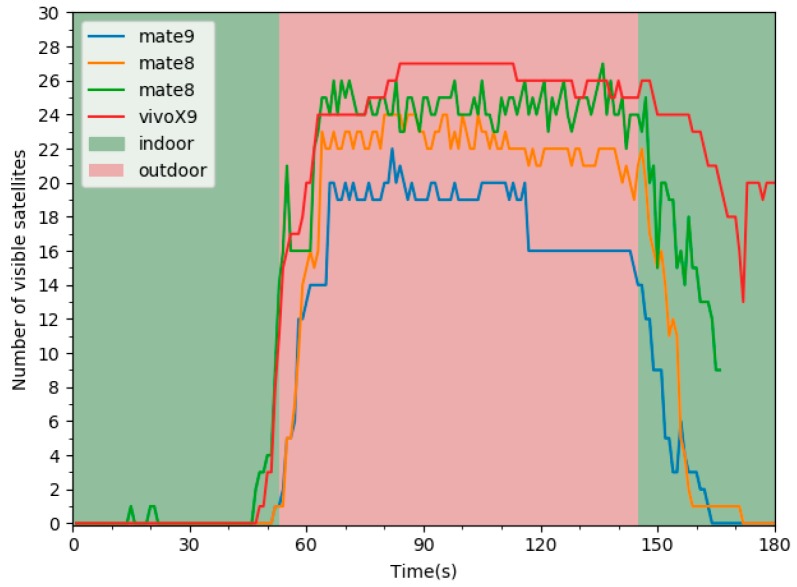
The changes in the number of visible satellites under different devices when switching between indoor and outdoor scenarios.

IO transitions are a continuous process. It is worth noting that time sequence features contain more useful information. Figure 8 demonstrates the changes in the number of visible satellites under different devices when switching between indoor and outdoor scenarios. Due to the weak satellite signal in the indoor scenarios, the number of satellites detected by the terminals is low. When the smartphones switch from indoors to outdoors, the number of visible satellites increases rapidly. However, when switching from outdoors to indoors, it can be seen that the satellite is in the tracking status, and the receiver conducts targeted integration and other processing on the tracked satellites, the terminal can still detect these satellites within a certain period.

To mitigate the effects of signal diversity in a different environment and alleviate the influences of device heterogeneity on GNSS measurements, we utilized the number of satellites at the current time to calculate the weight of the change of the number of satellites. Inspired by the changes in the number of visible satellites, we defined ${\mathrm{GS}\_\mathrm{Num}\_w}_{t_{1}{-t}_{2}}$ as follows:

(9) $${\mathrm{GS}\_\mathrm{Num}\_w}_{t_{1}{-t}_{2}}=\frac{cnt_{t_{1}}-cnt_{t_{2}}}{cnt_{t_{1}}}$$

where $cnt_{t_{1}}$ denotes the number of satellites at current time *t*, $cnt_{t_{2}}$ denotes the number of satellites at time *t*-*k*. *k* denotes the window size (e.g., 1, 2, 3). In the experiment, we compared the influence of the feature under different window size on indoor and outdoor detection.

#### 2.4.4. Visible Satellite CNR vary ratio

**Figure 9:**
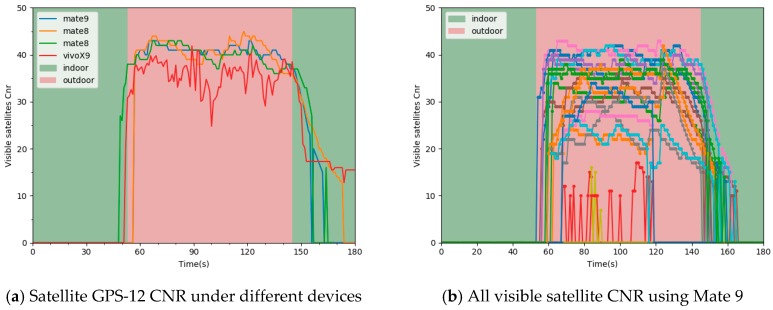
The variation of satellite CNR when an indoor and outdoor transition in the same scenario.

Visible satellite CNR vary ratio is also an essential feature in time sequence. Figure 9a shows the variation trend of GPS-12 CNR with different devices when IO transition under the same scenario. Figure 9b shows the trend of all visible satellite CNR using Mate 9. The variation trend of satellite CNR is close to that of the number of satellites in Figure 8 when IO transition. It is worth noting that the satellite CNR varies widely over time in both outdoor and indoor scenarios. It is not advisable to extract features by relying on the variation trend of single satellite CNR.

To avoid the influence mentioned above of the variation trend of single satellite CNR, we mined and utilized the variation trend of all visible satellites as features in time sequence. We defined a function $f_{\left(x,y\right)}$ that represents the variation of the satellite CNR in two moments as follows:

(10) $$f_{\left(x,y\right)}=sign\left(cnr_{i}^{x}-cnr_{i}^{y}\right)|i=0,1,\cdots ,n$$

where $cnr_{i}^{x}$ denotes the satellite CNR at current time t, $cnr_{i}^{y}$ denotes the satellite CNR at time *t-k*. *k* denotes the window size (e.g., 1, 2, 3). In the experiment, we compared the influence of the feature under different window size on indoor and outdoor detection.

We extracted the variation ratio of all visible satellite in $G_{u}$ as features. We defined the formulas for the descending ratio P(down), rising ratio P(up) and flat ratio P(hold) of all visible satellite CNR at different times as Equations (11), (13) and (14), respectively.

(11) $$P(\mathrm{down})=\frac{n-\sum_{i=1}^{n}sign\left(f_{\left(x,y\right)}+1\right)}{n}$$

(12) $$P_{1}=\frac{\sum_{i=1}^{n}abs\left(sign\left(f_{\left(x,y\right)}\right)\right)}{n}$$

(13) $$P(\mathrm{up})=P_{1}-P(\mathrm{down})$$

(14) P(hold)=1−P(down)−P(up)

#### 2.4.5. Statistical Features

**Figure 10:**
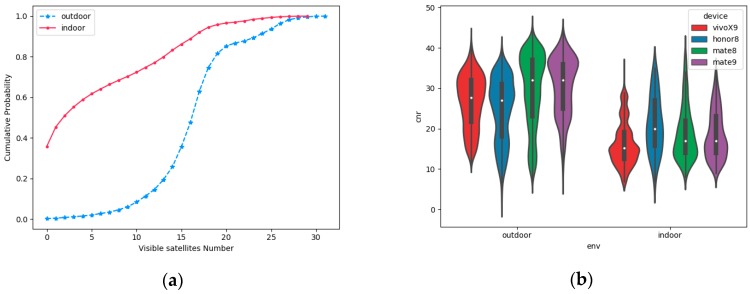
The statistical feature of GNSS measurements in indoor and outdoor scenarios. (**a**) The cumulative distribution probability of the number of visible satellites using different smartphones in an indoor and outdoor environment; (**b**) The violin plot of visible satellite CNR using four types of smartphone in the same scenario.

The number of satellites and the distribution of satellite CNR is different in indoor and outdoor scenarios. As shown in Figure 10a, the cumulative probability of indoor scenarios is close to 0.7 when the number of visible satellites is under 8, while the cumulative probability of outdoor scenarios is lower than 0.05 when the number of visible satellites is under 8. Figure 10b shows that the satellite CNR collected by different devices in the same scenario is different, but the distribution density tends to be consistent. In other words, the statistical data of the number of satellites and CNR are effective features to distinguish complex indoor and outdoor scenarios.

To utilize more descriptive information under sliding windows of different lengths, we not only regard the number of the visible satellite as a feature, but also extract Mean, Variance, Std, Min, Max, Median, Range, InterQuartile Range, Skewness and Kurtosis of satellite CNR from G and $G_{p}$ as features. Furthermore, we consider the mean of PDoP, HDoP and VDoP in $S_{u}$. 

### 2.5. Classification Model

#### 2.5.1. Single Classification Model

In the training phase, we try to train different model using a variety of machine learning algorithms such as RF [34], SVM [35], Adaptive Boosting (AdaBoost) [20], XGBoost (XGB) [36] and LightGBM (LGB) [37]. The training data contains GNSS information features extracted from different sliding window sizes. In the testing phase, we evaluated different classifier for indoors/outdoors detection.

#### 2.5.2. Classification Model Based on Stacking Ensemble

**Figure 11:**
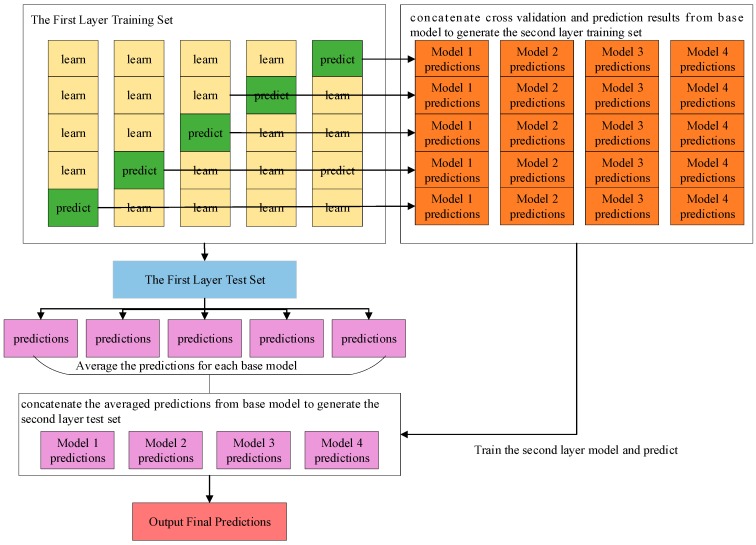
A framework of the model based on stacking ensemble.

Stacking is a model ensembling technique, which uses the initial training data to learn some base learners and uses the predicted results of these learners as a new training set to generate a new model. In general, the stacked model outperforms each of the individual models due to its smooth nature and ability to highlight each base model where it performs best and discredit each base model where it performs poorly. As shown in Figure 11, we used a 2-layer stacking model for training. XGBoost, LightGBM, AdaBoost and Random Forest are used to train the base model in the first layer to generate the train set and the test set for the second layer. Logistic regression is employed to output the final prediction in the second layer.

#### 2.5.3. Hidden Markov Model

HMM is a model based on probability statistics. In this paper, we use the first-order HMM, which assumes the current scenario state is only affected by the previous state. The probabilities of each state at each epoch can be inferred by the Viterbi algorithm [38] from the observations sequence. In general, an HMM comprises five elements as follows:

1) The state space *S* that consists of two hidden states: indoor and outdoor, which are denoted as *S*_0_ and *S*_1_.
2) The set of observations at each epoch refers to the predicted result of the supervised model on the testing set.
3) The matrix of state transition probabilities was set by prior experience. Table 2 lists the values of transition probability.
4) The matrix of emission probabilities refers to normalized confusion matrix of the predicted results of the supervised model on the testing set. Table 3 shows the emission probabilities of each state to each feature.
5) The initial state *X*_1_ probabilities set as follows:
  (15) $$\begin{array}{l} P(X_{1}=S_{0})=0.5\\ P(X_{1}=S_{1})=0.5 \end{array}$$

## 3. Results

### 3.1. Experimental Setup

#### 3.1.1. Data Collection

We conducted all our experiments under a variety of weather conditions, including sunny, cloudy and hazy days in the Beijing urban area. Volunteers collected GNSS data in 58 scenarios, including indoors, outdoors and IO transitions on campus, and in a shopping mall, restaurant, office building, pedestrian street, an overpass and a residential area, with four different types of phones (Huawei Mate 8, Huawei Mate 9, Huawei Honor 8, Vivo X9) within a month. The mobile phone system version was Android 7.0 or above.

Our volunteers move naturally with their phones held in front of their chest when they collected data in the complex indoor and outdoor scenarios. The only constraint was to modify the ground-truth IO label when they switch between indoor and outdoor scenarios. Especially, we collected data from deep indoors, shallow indoors, semi-open outdoors and open outdoors to provide the credibility of IO detection in complex scenarios. A group of collected data was divided into three categories: indoor data collection, outdoor data collection, and IO transition data collection. The data collection time of each log file was more than 1 minute.

#### 3.1.2. Dataset Segmentation

To ensure the performance on the validation set approximates to it on the test set, we split the dataset by mobile phone serial number and scenario. To train the universal I/O classification model, we selected a dataset_0 that contained 118,432 data items including 15 indoor and outdoor scenes and 18 indoor and outdoor switching scenes. We hope that a classification model can accurately identify both pure indoor and outdoor scenes and achieve brilliant performance in indoor and outdoor switching scenes. 

In order to assess the performance of our model in different scenarios, we selected dataset_1, dataset_2, dataset_3 and dataset_4 to comprehensively evaluate our classification model from the aspects of recognition accuracy and switching delay. Especially, dataset_1 and dataset_3 were used to evaluate the performance of IO detection. Dataset_2 and dateset_4 were used to evaluate the performance of IO transitions detection. The data in these four test sets are untrained. The scenario of dataset_1 and dataset_2 are the same as the IO scenarios and I/O switching scenarios of dataset_0 respectively, while dataset_3 and dataset_4 were collected in the new scenario. Dataset_ 3 and dataset_ 4 would be used to evaluate our model’s ability to adapt to the new test environment.

Dataset_1 was used to evaluate the recognition performance of indoor and outdoor scenes that had been trained in the training stage. 17,722 data items were collected in these indoor and outdoor scenes in dataset_1. Dataset_2 was used to evaluate the recognition performance of I/O switching scenes that have been trained in the training stage. 31,290 data items were collected in these I/O switching scenes in dataset_2. Dataset_3 was used to evaluate the recognition performance of indoor and outdoor scenes that had been untrained in the training stage. 17,199 data items were collected in these indoor and outdoor scenes in dataset_3. Dataset_4 was used to evaluate the recognition performance of I/O switching scenes that have been trained in the training stage. 11,218 data items were collected in these I/O switching scenes in dataset_4. Table 4 presents the detail distribution of five datasets. 

### 3.2. Refined Classifier Performance Evaluation

#### 3.2.1. Accuracy Evaluation of Different Features and Models

We evaluated the accuracy of five single classification models including RF, SVM, AdaBoost, XGB and LGB, as well as a classification model based on stacking ensemble and a stacking model with HMM in four datasets. The detection accuracy was compared under different kinds of features including only statistical features in different sliding window length k, only spatial geometry distribution features; only time sequence features in different sliding window length k and a combination of the above features. Table 5, Table 6, Table 7 and Table 8 show the comparison results on Dataset_1, Dataset_2, Dataset_3 and Dataset_4, respectively.

From Table 5, Table 6, Table 7 and Table 8, we obtained the following conclusions:

- The stacking model with HMM performed best on four datasets overall. The accuracy of dataset_1, dataset_2, dataset_3 and dataset_4 were 0.9911, 0.9453, 0.9702 and 0.9280, respectively. Furthermore, SVM obtained the lowest accuracy in almost all experiments, the accuracy of LightGBM was superior to other single classification model and slightly lower than the stacking model with HMM in most experiments.
- The different sliding window size influenced the accuracy of different datasets. For statistical features in four datasets, using the statistical features of the current time (k = 1) can obtain higher accuracy, while for the time sequence features, the bigger the window size was, we obtained the higher accuracy.
- The accuracy of only use statistical features on Dataset_1, Dataset_2, Dataset_3 and Dataset_4 was 0.9893, 0,9344, 0.9319 and 0.9185, respectively. However, in the vast majority of cases, the accuracy was under 0.91 when only used time sequence features. The accuracy reached 0.9662 on Dataset_1 when only used spatial geometry distribution features, while in other datasets, the accuracy was less than 0.81. This result showed that statistical features play the most crucial role in IO detection. Since the number of features contained in spatial geometry distribution and time sequence was small, only using these two kinds of features lead to low accuracy.
- It was worth noting that spatial geometry distribution features and time sequence features improved accuracy more than 0.01 on Dataset_2 and Dataset_4. Furthermore, the improved accuracy of the two kinds of features mentioned above was closed to 0.04 on Dataset_3. Therefore, spatial geometry distribution features and time sequence features contributed to IO detection.
- In indoor and outdoor scenarios, the optimal accuracy of Dataset1 was 0.0209 higher than that of Dataset3 using the same kinds of features. In IO transition scenarios, the optimal accuracy of Dataset2 was 0.0173 higher than that of Dataset4 using the same kinds of features. While the accuracy of our proposed algorithm in the new test environment was lower than that in the environment where collected training data, the overall accuracy in dataset_3 and dataset_4 were more than 0.9280, that means the proposed algorithm robust among different complex environments (non-sampled environments).

#### 3.2.2. Feature Importance Analysis

**Figure 12:**
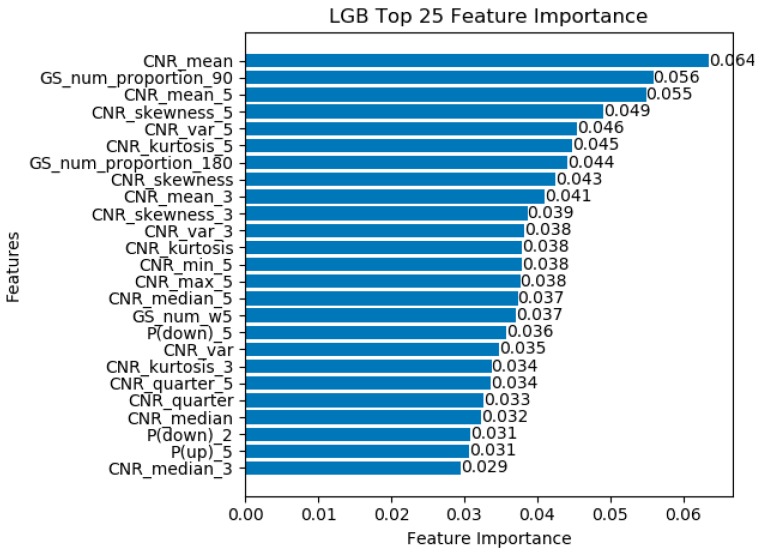
Normalized sorted features importance in LightGBM training process.

According to the accuracy on all test datasets, LightGBM performed best in all single classification model. To intuitively reflect the different kinds of features used in the LightGBM classifier training stage, we focus on the top 25 features importance. Figure 12 shows the ranking of feature importance.

As shown in Figure 12, we found that the mean of satellite CNR feature at a current time, GS_num_proportion_90 and the skewness of satellite CNR played the most three crucial roles in the training stage. The statistical features play the most important role in the training phase that accounts for 67.97% of the top 25 features due to a large number of statistical features were extracted. It is worth noting that the time sequence related features account for 17.78% and spatial geometry distribution related features account for 14.25%, also reflecting the effectiveness of these two kinds of related features.

#### 3.2.3. Transition Delay

**Figure 13:**
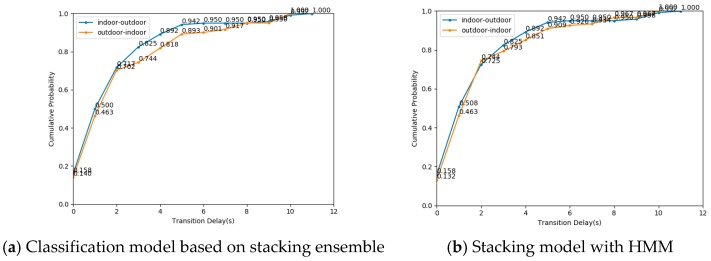
The cumulative distribution probability of transition delay using different algorithms on dataset_2.

In this section, we evaluated the IO transition delay based on the proposed IO detection algorithm. According to the detection accuracy in Table 5, we focused on the model based on stacking ensemble and a stacking model with HMM. We first verified the performance of the mentioned above two algorithms on dataset_2. Figure 13 shows the cumulative probability of transition delay using different algorithms on dataset_2. Under the classification model based on stacking ensemble, the transition delay from indoor to outdoor is lower than that from outdoor to indoor. There is little difference between indoor to outdoor transition delay and outdoor to indoor transition delay by a stacking model with HMM. The cumulative distribution probability of the stacking model and a stacking model with HMM algorithm of switching delay in 3 s reached more than 80%. 

**Figure 14:**
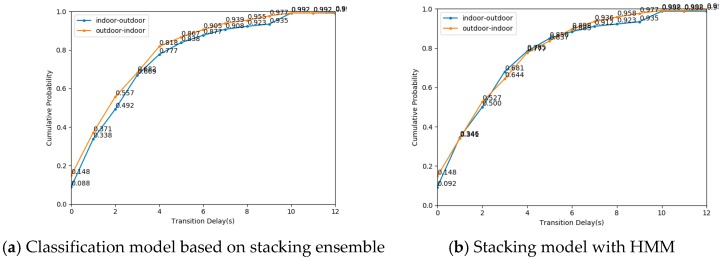
The cumulative distribution probability of transition delay using different algorithms on dataset_4.

We also evaluated the transition delay of our algorithm in new scenarios. Figure 14 shows the cumulative probability of transition delay using different algorithms on Dataset_4. There is little difference between indoor to outdoor transition delay and outdoor to indoor transition delay under these two algorithms. The cumulative probability of two algorithms of transition delay in 4 s reached more than 80%. The transition delay of Dataset_4 was slightly higher than that of Dataset_2.

The above experiments show that our algorithm meets the requirement of quickly detect IO transition. Furthermore, the performance of our algorithm in transition delay decreases slightly in the new environment using new smartphones. Meanwhile, the HMM model has no obvious effect on reducing transition delay.

### 3.3. Performance Comparison with other Algorithms

We compared the proposed algorithm with other state-of-the-art IO detection methods (SatProbe [21] and Gao et al. [22]). SatProbe only used the number of GPS visible satellites as a more direct indicator of IO status (indoor, semi-outdoor and outdoor). In SatProbe, if the detected satellite count is no more than 2, then the IO status is indoor. If there are six or more satellites in view, then the IO status is outdoor. For situations between, if the ambiguity persists, the IO status is determined to be semi-outdoor. Gao et al. extracted the number of visible satellites that CNR more than 25 dB-Hz (numCNR_25_) and the sum of all visible satellites CNR that more than 25 db-Hz (sumCNR_25_) based on the availability and strength of GNSS signals (GPS and GLONASS) as features. Then, a Hidden Markov Model was used to infer the current environment types (indoors, intermediate and outdoors) according to those extracted features.

#### 3.3.1. Evaluation of Visible GPS Satellite Number Algorithm

**Figure 15:**
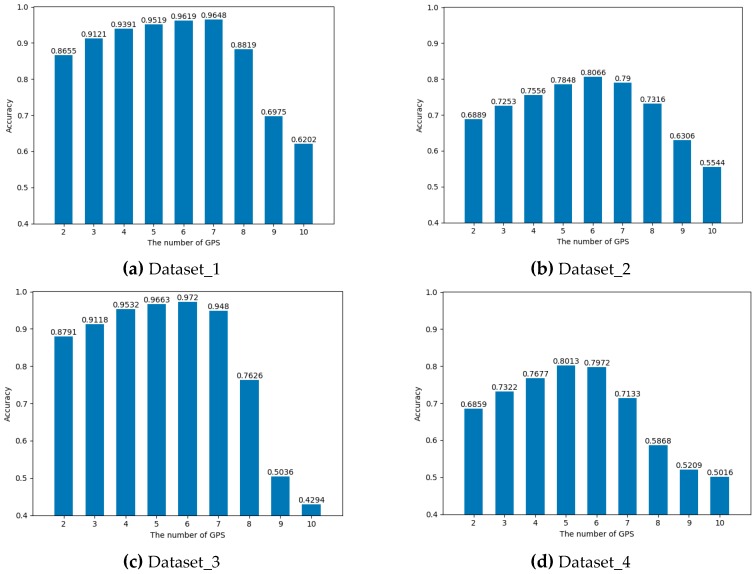
The accuracy of indoor and outdoor detection using different GPS number as a threshold on four datasets.

However, there is no ambiguity state like semi-outdoors or intermediate in our datasets. We focus on these complex scenarios, but the final detection states only include indoors and outdoors. Therefore we did not use the same experimental parameters. For SatProbe, we used different visible GPS satellite numbers as thresholds for IO detection to obtain the optimal performance of the algorithm in our datasets. Figure 15 shows the experimental results.

As shown in Figure 15, when the threshold value set to 6, SatProbe achieved the highest accuracy in Dataset_2 (0.8066) and Dataset_3 (0.9720), and the second highest accuracy in Dataset_1 (0.9619) and Dataset_4 (0.7972). Since lack of fuzzy state as semi-outdoor, only depending on the number of GPS satellites, thus resulting in the performance of IO transition is very poor. 

**Figure 16:**
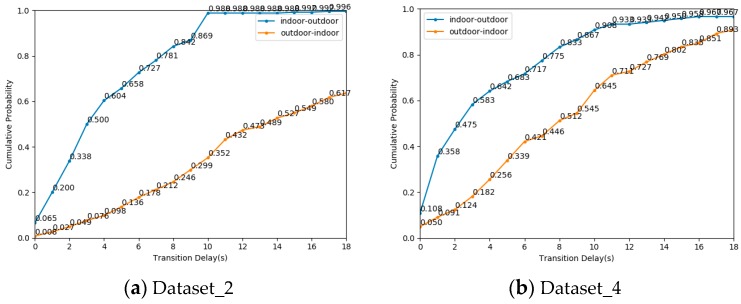
The cumulative distribution probability of transition delay using SatProbe algorithm on Dataset_2 and Dataset_4.

Next, we evaluated the IO transition delay when the threshold is 6. Figure 16 described the cumulative distribution probability of time delay on Dataset_2 and Dataset_4. To achieve more than 80% cumulative probability of indoor to outdoor transition on Dataset_2 and Dataset_4, the IO transition delay must be greater than 8 s and 8 s, respectively. To achieve more than 80% cumulative probability of outdoor to indoor transition on Dataset_2 and Dataset_4, the IO transition delay must be greater than 18 s and 14 s, respectively. It is difficult to detect the transition from outdoor scenes to indoor scenes in a short time only depending on the number of satellites. Such a long transition delay is difficult to accept for seamless indoor and outdoor positioning.

#### 3.3.2. Evaluation of GNSS Signals Algorithm

**Figure 17:**
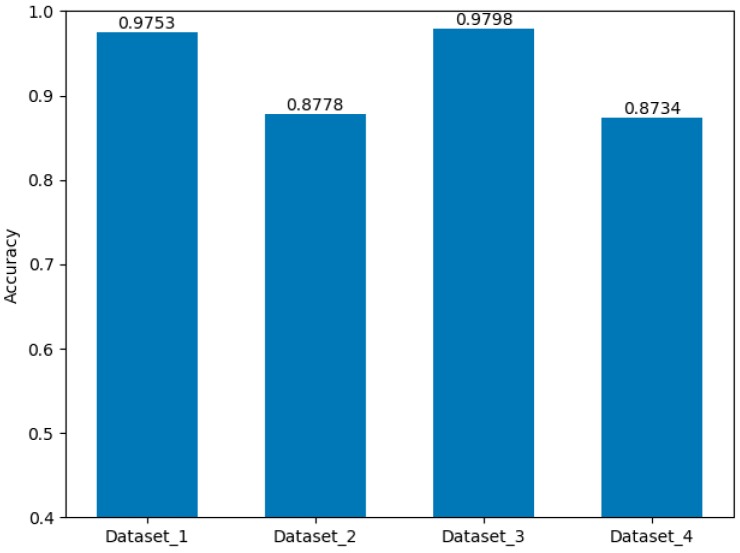
The accuracy of indoor and outdoor detection using algorithm [22] on four datasets.

This algorithm also divided the detection status into three types, which was similar to the definition of a fuzzy intermediate state of SatProbe. Hence, we modified the mean and variance of the Gaussian distribution in the emission matrix of the algorithm according to the statistical data of the features extracted by the algorithm from the training set. Furthermore, we adjusted the transition probabilities of HMM. We evaluated the accuracy of the algorithm on four datasets. As shown in Figure 17, the accuracy of this algorithm in Dataset_2 (0.8778) and Dataset_4 (0.8774) is nearly 0.10 lower than that in Dataset_1 (0.9753) and Dataset_3 (0.9798). Similarly, the IO detection accuracy of IO transition regions is lower than indoor and outdoor scenarios.

**Figure 18:**
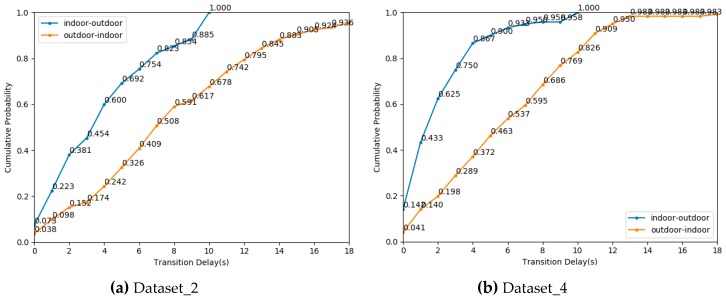
The cumulative probability of transition delay using algorithm [22] on Dataset_2 and Dataset_4.

Next, we evaluated the IO transition delay on Dataset_2 and Dataset_4. Figure 18 showed the cumulative distribution probability of time delay on Dataset_2 and Dataset_4. To achieve more than 80% cumulative probability of indoor to outdoor transition on Dataset_2 and Dataset_4, the IO transition delay must be greater than 7 s and 4 s, respectively. To achieve more than 80% cumulative probability of outdoor to indoor transition on Dataset_2 and Dataset_4, the IO transition delay must be greater than 12 s and 10 s, respectively. The transition efficiency of the algorithm [22] is better than that of SatProbe. However, 12 s is still unacceptable.

#### 3.3.3. Overall Performance Comparison

In this section, we evaluated the performance of our proposed algorithm and the two comparison algorithms on four datasets as a whole. The detail experiments results are shown in Table 9. 

From Table 9 we obtain the following main conclusions:

- In indoor and outdoor scenes, three indoor and outdoor recognition algorithms have high accuracy. Compared with the other two algorithms, the accuracy of our detection algorithm was 0.0158 higher than that of the other two algorithms in Dataset_1, and algorithm [22] was 0.0096 higher than our algorithm in Dataset_3.
- In indoor and outdoor transition scenes, our detection algorithm is superior to the other two algorithms regarding accuracy and transition delay. The detection accuracy of our algorithm on Dataset_2 and Dataset_4 is 0.1387 and 0.1308 higher than SatProbe. The indoor to outdoor transition delay of out algorithm on Dataset_2 and Dataset_4 is 5 s and 4 s faster than that of SatProbe. Furthermore, the outdoor to indoor transition delay of our algorithm on Dataset_2 and Dataset_4 is 15 s and 10 s faster than that of SatProbe.
- The detection accuracy of our algorithm on Dataset_2 and Dataset_4 is 0.0675 and 0.0546 higher than algorithm [22]. The indoor to outdoor transition delay of our algorithm on Dataset_2 is 4 s faster than that of the algorithm [22]. Furthermore, the outdoor to indoor transition delay of our algorithm on Dataset_2 and Dataset_4 is 9 s and 6 s faster than that of the algorithm [22].
- From the above data, we can find that the indoor and outdoor detection algorithm we proposed can accurately identify the indoor and outdoor state in a complex environment. Especially, our algorithm enables the ability to quickly detect the indoor and outdoor transition in 4 s with a probability of more than 0.8 that other algorithms cannot.

### 3.4. Algorithm Complexity Evaluation

To measure the complexity of the proposed algorithm, we compared the training time cost of each classification model running on a computer with Intel E5-2680 CPU and 64GB memory. Table 10 lists the time cost of training different classification models.

Since we only extracted low-dimensional features from GNSS measurements, it did not require expensive training time cost. The training speed of LightGBM was superior to other classification models. Furthermore, the model ensemble process required an abundant computational procedure to train multiple models, and it was broadly in line with what we expected. We comprehensively considered the accuracy and training time, LightGBM used the minimum training time to obtain a higher prediction accuracy in five single classification models. While the classification model based on stacking ensemble with HMM obtained the optimal accuracy on Dataset_2 and Dataset_4, its performance on training was the worst due to the stacking model cost too much time. 

## 4. Conclusions

In this paper, we have proposed a fast indoor/outdoor transition detection algorithm based on machine learning without any infrastructure. We extracted statistical and time sequence features under different sliding window lengths and spatial geometry distribution features from GNSS measurements. To evaluate IO detection accuracy and transition delay of the proposed algorithm, we conducted experiments in the complex indoor and outdoor environments in urban Beijing. The evaluation results demonstrate that the IO detection accuracy was 99.11% in indoor and outdoor scenarios where we have collected data and 97.02% in new indoor and outdoor scenarios. Furthermore, IO detection accuracy was 94.53% in indoor and outdoor transition scenarios where we have collected data and the probability of switching delay within 3 s exceeds 80%. In the new scenarios, IO detection accuracy was 92.80% and the probability of switching delay within 4 s exceeds 80%. The proposed algorithm outperforms other existing IO detection methods and satisfies the requirement of indoor and outdoor seamless navigation and positioning.

In our future work, we will further improve detection accuracy, shorten the switching delay and expand the scale of the experiment. Also, we will consider extracting more universality features to eliminate device heterogeneity. Furthermore, we will design an indoor and outdoor seamless navigation and positioning system based on smartphone for pedestrians.

## 5. Patents

The proposed algorithm is applying for a patent and now has been handed over to the agency.

## Figures and Tables

**Table 1:** The list of considered features for our classifiers.

Category		Features	Description
**Spatial Geometry Distribution**	**Visible satellite geometry topology**	Az_dtb_vector	A 36-dimensional vector d represents the azimuth distribution of the satellite
		Az_dtb_proportion	Satellite azimuth distribution proportion
		GS_num_proportion_90	The proportion of the number of satellites within the range of 90° of azimuth
		GS_num_proportion_180	The proportion of the number of satellites within the range of 180° of azimuth
	**DoP for positioning satellite**	PDoP, HDoP, VDoP	To measure the influence of the spatial geometric distribution of observation satellites on the positioning accuracy
**Time Sequence**	**Number of visible satellites vary in weight**	${\mathrm{GS}\_\mathrm{Num}\_w}_{t_{1}{-t}_{2}}$	The number of satellites change ratio from time t_2_ to time t_1_
	**Visible satellite CNR vary ratio**	${P(\mathrm{down})}_{t_{1}{-t}_{2}}$	Satellite CNR in collection G_u_ down ratio from time t_2_ to time t_1_
		${P(\mathrm{hold})}_{t_{1}{-t}_{2}}$	Satellite CNR in collection G_u_ hold ratio from time t_2_ to time t_1_
		${P(\mathrm{up})}_{t_{1}{-t}_{2}}$	Satellite CNR in collection G_u_ up ratio from time t_2_ to time t_1_
**Statistical**	**Collection** G	GS_Num	The number of satellites at the current time
		CNR_mean, CNR_var, CNR_std, CNR_min, CNR_max, CNR_median, CNR_range, CNR_iqr, CNR_ ske, CNR_kur	Mean, Variance, Std, Min, Max, Median, Range, InterQuartile Range, Skewness, Kurtosis of satellite CNR in G
	**Collection** $G_{p}$	GS_Num_k	The number of satellites in $G_{p}$ under different sliding window lengths *k*
		CNR_mean_k, CNR_var_k, CNR_std_k, CNR_min_k, CNR_max_k, CNR_median_k, CNR_range_k, CNR_iqr_k, CNR_ ske_k, CNR_kur_k	Mean, Variance, Std, Min, Max, Median, Range, InterQuartile Range, Skewness, Kurtosis of satellite CNR in $G_{p}$ under different sliding window lengths *k*
	**Collection** $S_{u}$	PDoP_mean, HDoP_mean, VDoP_mean	Mean of PDoP, VDoP, HDoP in $S_{u}$

**Table 2:** Transition probabilities of HMM.

Status	Indoors	Outdoors
**Indoors**	0.8	0.2
**Outdoors**	0.2	0.8

**Table 3:** Emission probabilities of HMM.

Status	Indoors	Outdoors
**Indoors**	$\frac{TP}{TP+TN+FP+FN}$	$\frac{FN}{TP+TN+FP+FN}$
**Outdoors**	$\frac{FP}{TP+TN+FP+FN}$	$\frac{TN}{TP+TN+FP+FN}$

**Table 4:** The detail distribution of the datasets we segment.

	Number of Scenarios	Data Items	Device	Scenarios Category
Dataset_0	33	118432	Mate 8-1, Mate 8-2, Honor 8-1, Mate 9-1, Mate 9-2, Vivo X9-1	Indoor, Outdoor, IO Transition
Dataset_1	15	17722	Mate 8-1, Mate 8-2, Honor 8-1, Mate 9-1, Mate 9-2, Vivo X9-1	Indoor, Outdoor
Dataset_2	18	31290	Mate 8-1, Mate 8-2, Mate 8-1, Mate 9-1, Mate 9-2, Vivo X9-1	IO Transition
Dataset_3	10	17199	Mate 8-3, Vivo X9-2, Mate 9-3, Honor 8-2	Indoor, Outdoor
Dataset_4	15	11218	Mate 8-3, Vivo X9-2, Mate 9-3, Honor 8-2	IO Transition

**Table 5:** Indoor/Outdoor detection accuracy using different features and models on Dataset_1.

Model	S1	S3	S5	SD	TS2	TS3	TS5	S&SD&TS
RF	0.9864	0.9847	0.9851	0.9646	0.8813	0.8932	0.9019	0.9900
SVM	0.9787	0.9756	0.9763	**0.9662**	0.8557	0.8588	0.8594	0.9852
AdaBoost	0.9875	0.9856	0.9853	0.9439	0.8818	0.8929	0.9009	0.9909
XGB	0.9858	0.9841	0.9848	0.9590	0.8919	0.8980	0.9072	0.9900
LGB	0.9889	**0.9873**	0.9857	0.9593	0.8822	0.9027	0.9102	0.9902
Stacking	0.9870	0.9848	0.9858	0.9589	0.8894	0.8993	0.9023	0.9908
Stacking &HMM	**0.9893**	0.9863	**0.9861**	0.9593	**0.8935**	**0.9109**	**0.9132**	**0.9911**

Sk denotes only use statistical features when window size equals k. SD denotes only use spatial geometry distribution features. TSk denotes only use time sequence features when window size equals k. S&SD&TS denotes jointing statistical, spatial geometry distribution and time sequence features under different window size. The numbers in bold and highlighted represent the highest accuracy.

**Table 6:** Indoor/Outdoor detection accuracy using different features and models on Dataset_2.

Model	S1	S3	S5	SD	TS2	TS3	TS5	S&SD&TS
RF	0.9312	0.9312	0.9290	0.8047	0.8401	0.8527	0.8661	0.9451
SVM	0.9155	0.9082	0.9032	0.8085	0.7784	0.8101	0.8228	0.9350
AdaBoost	0.9299	0.9296	0.9283	0.7877	0.8411	0.8540	0.8673	0.9413
XGB	0.9323	0.9303	0.9290	0.8106	0.8456	0.8620	0.8744	0.9431
LGB	0.9342	0.9310	0.9290	0.8041	0.8437	0.8621	0.8722	0.9446
Stacking	0.9320	0.9313	0.9293	0.8043	0.8484	0.8620	0.8738	0.9435
Stacking &HMM	**0.9344**	**0.9332**	**0.9295**	**0.8132**	**0.8501**	**0.8644**	**0.8752**	**0.9453**

**Table 7:** Indoor/Outdoor detection accuracy using different features and models on Dataset_3.

Model	S1	S3	S5	SD	TS2	TS3	TS5	S&SD&TS
RF	0.9256	0.9247	0.9267	0.7923	0.8394	0.8572	0.8669	0.9632
SVM	0.9273	0.9238	0.9300	**0.8186**	0.8657	0.8683	0.8673	0.9155
AdaBoost	0.9290	0.9263	0.9282	0.7796	0.8378	0.8558	0.8652	0.9689
XGB	0.9323	0.9278	0.9303	0.7938	0.8577	0.8740	0.8777	0.9648
LGB	**0.9319**	0.9263	0.9287	0.7960	**0.8624**	0.8762	0.8760	**0.9710**
Stacking	0.9290	0.9285	0.9301	0.7952	0.8495	0.8671	0.8725	0.9688
Stacking &HMM	0.9313	**0.9296**	**0.9311**	0.7966	0.8573	**0.8772**	**0.8846**	0.9702

**Table 8:** Indoor/Outdoor detection accuracy using different features and models on Dataset_4.

Model	S1	S3	S5	SD	TS2	TS3	TS5	S&SD&TS
RF	0.9168	0.9120	0.9096	0.7968	0.8264	0.8343	0.8412	0.9255
SVM	0.9039	0.8977	0.8922	0.8186	0.7836	0.7958	0.7906	0.9131
AdaBoost	0.9110	0.9131	0.9120	0.7738	0.8259	0.8365	0.8405	0.9245
XGB	0.9168	0.9156	0.9096	0.7938	0.8404	0.8471	**0.8533**	0.9251
LGB	0.9179	**0.9202**	**0.9138**	**0.7969**	**0.8415**	**0.8486**	0.8510	0.9258
Stacking	0.9172	0.9151	0.9120	0.7922	0.8367	0.8433	0.8516	0.9268
Stacking &HMM	**0.9179**	0.9163	0.9132	0.7930	0.8396	0.8458	0.8527	**0.9280**

**Table 9:** Accuracy and transition delay evaluation of different algorithms on four datasets.

Dataset	Algorithm	Accuracy	Indoor to Outdoor transition delay when the cumulative probability reaches 0.8	Outdoor to Indoor Transition delay when the cumulative probability reaches 0.8
**Dataset_**1	Proposed	0.9911	No transition delay	No transition delay
	SatProbe	0.9619		
	Gao et al. [22]	0.9753		
**Dataset_**2	Proposed	0.9453	3s	3s
	SatProbe	0.8066	8s	18s
	Gao et al.	0.8778	7s	12s
**Dataset_**3	Proposed	0.9702	No transition delay	No transition delay
	SatProbe	0.9720		
	Gao et al.	0.9798		
**Dataset_**4	Proposed	0.9280	4s	4s
	SatProbe	0.7972	8s	14s
	Gao et al.	0.8734	4s	10s

**Table 10:** The comparison of different algorithm training time.

Model	Training Time(s)
RF	20
SVM	819
AdaBoost	356
XGB	9
LGB	2
Stacking Based Model Ensemble	1133
Stacking Model & HMM	1135
